# Windscapes and olfactory foraging in a large carnivore

**DOI:** 10.1038/srep46332

**Published:** 2017-04-12

**Authors:** Ron R. Togunov, Andrew E. Derocher, Nicholas J. Lunn

**Affiliations:** 1 Department of Biological Sciences, University of Alberta, Edmonton, AB T6G 2E9, Canada; 2 Wildlife Research Division, Science & Technology Branch, Environment and Climate Change Canada, CW-422, Biological Sciences Building, University of Alberta, Edmonton, AB T6G 2E9, Canada

## Abstract

The theoretical optimal olfactory search strategy is to move cross-wind. Empirical evidence supporting wind-associated directionality among carnivores, however, is sparse. We examined satellite-linked telemetry movement data of adult female polar bears (*Ursus maritimus*) from Hudson Bay, Canada, in relation to modelled winds, in an effort to understand olfactory search for prey. In our results, the predicted cross-wind movement occurred most frequently at night during winter, the time when most hunting occurs, while downwind movement dominated during fast winds, which impede olfaction. Migration during sea ice freeze-up and break-up was also correlated with wind. A lack of orientation during summer, a period with few food resources, likely reflected reduced cross-wind search. Our findings represent the first quantitative description of anemotaxis, orientation to wind, for cross-wind search in a large carnivore. The methods are widely applicable to olfactory predators and their prey. We suggest windscapes be included as a habitat feature in habitat selection models for olfactory animals when evaluating what is considered available habitat.

Foraging efficiency, energy acquisition per unit time, is central to an animal’s fitness. Natural selection favours behaviours that maximize energy intake while minimizing foraging time^1^. Foraging behaviour by predators can be classified into two broad classes: ambush predation and active search predation^2^. For ambush predation, fitness is largely determined by habitat selection^3^. For active search predation, studies have expounded the significance of duration of patch use^4^ and prey selection^5^, however, research on optimal search strategies among large carnivores remains sparse^6^. Search strategies are especially important for success at large scales^6^.

Olfactory search is common for foraging carnivores^7^, beginning with identifying the presence of prey through odour detection, followed by prey localization^8^. The optimal olfactory search and localization strategies are largely dependent on the structure of odour plumes and patterns of odour dispersion^8^. In turbulent flow, odours are concentrated into meandering filaments, which occur at higher densities approaching the odour source^9^,^10^. Time-averaged odour concentration can be described by the Gaussian dispersion model, whereby the maximum odour concentration is along the horizontal axis in the direction of the wind, and mean concentration follows a normal distribution laterally and vertically^10^. Gaussian dispersion, however, does not accurately describe instantaneous odour distribution and cannot be assumed for plume-tracking at small spatiotemporal scales^11^. Nonetheless, Gaussian dispersal performs well for behaviours related to longer-term exposure, such as large-scale olfactory search^10^. During olfactory search, movement should lead to position a predator where it is most likely to encounter an odour filament. Once detected, the predator should move upwind from the location of detection to localize the source, with successful location resulting in a predation attempt or a kill. For olfactory search at large scales or in steady winds, traveling cross-wind is the optimal path for encountering an odour plume^11^,^12^. However other search strategies such as up-wind, down-wind, Lévy walk, and correlated random walk may be effective depending on patterns of odour dispersion^8^,^13^. Anemotaxis, orientation relative to wind, is well documented among insects and, more recently, among some birds, which travel cross-wind when searching for an odour and upwind when localizing the source^14^,^15^,^16^, however, research on olfactory search among mammalian predators is sparse^17^.

Polar bears (*Ursus maritimus*) exhibit both ambush and active search predation strategies. Most polar bear foraging is confined to the sea ice, which also serves as the prime platform for travel and mating^18^. When hunting their primary prey, ringed seal (*Pusa hispida*) and bearded seal (*Erignathus barbatus*), polar bears may actively search for subnivean ringed seal pupping lairs or hauled-out seals, or ambush seals surfacing at breathing holes or along the floe edge^19^,^20^,^21^. Ringed seal densities can range from 0.46–1.6 seals/km^2^ and bearded seal densities can range from 0.0036–0.0229 seals/km^2^ in Hudson Bay^22^. Ringed seal breathing holes are spaced approximately 200 m or more apart^19^,^23^ and used by only 1–2 seals^19^,^22^ because clumped distributions may increase predation risk^19^. Ringed seals prefer land-fast ice and large floes with leads in pack ice^22^. Deformation of sea ice results in ridging that reach a mean peak height of 2 m, mean width of 12 m, and account for 10–40% of sea ice volume^24^. Therefore, vision is less effective for polar bears to locate prey from a distance. In addition to vision, polar bears exhibit strong responses to odours and often resort to olfactory search^19^,^20^,^23^,^25^ because winds can carry odours across the complex icescape. Olfactory bulb size is correlated with home range size among carnivores^7^, and polar bear home ranges are disproportionately large for their body size^26^ further suggesting reliance on olfaction. Although detection distance is hard to estimate in mammals^27^, estimates for polar bears suggest they may detect seal breathing holes up to 3 km away^28^. Additionally, olfactory predation is presumed to underlie ringed seal haul-out behaviour: they face downwind when hauled-out, enabling them to visually detect bears approaching from downwind and detect upwind bears by scent^29^. While odours associated with female ringed seals and their pups are unstudied, male ringed seals are known to produce pungent odours from facials glands^30^. Olfaction is likely also important in polar bear reproductive behaviour; males assess the reproductive status of females through their footprints and locate females by tracking their scent^31^. Females with cubs, may also use olfaction to avoid males due to risk of infanticide^32^,^33^.

Olfactory search is likely dependent on a number of factors including season, time of day, wind speed, and prey distribution. Polar bear populations inhabiting regions of seasonal sea ice are on land during the ice-free summer, move onto the ice during freeze-up, remain on the sea ice during winter and spring, and return to land during break-up. During summer, without access to their primary prey, terrestrial foraging is limited primarily to berries, seaweed, vegetation, bird eggs, and carrion^34^,^35^. Because these terrestrial food sources are not as energetically dense as seal fat^36^,^37^, polar bears prioritize energy conservation over energy acquisition and minimize unnecessary movement^32^,^36^,^38^,^39^. During freeze-up, bears may favour dispersion over immediate foraging to minimize intraspecific competition or, for females with dependent young, minimize risk of predation on their cubs^32^,^33^. Late winter and spring coincide with the peak in ringed seal and bearded seal pupping, when the majority of foraging takes place and bears enter hyperphagia^40^. During break-up, sea ice becomes increasingly dynamic and bears may favour travelling against the drift to maintain their relative position^41^,^42^ or move to shore as the cost of travelling exceeds the benefit of foraging^43^,^44^. With respect to time of day, olfactory search likely increases during periods of reduced visibility. For example, nocturnal moths rely more on olfaction to locate flowers than diurnal moths of the same subfamily, which rely more on visual search^45^.

Odour filament density and distribution within a plume are affected by wind speed. In slow winds, there may be insufficient directionality to assess the source of an odour, thus, bears may move independently of wind direction. Fast winds increase turbulence intensity and may disrupt odour plumes, which can impede odor localization^13^,^46^,^47^. Thus, cross-wind olfactory search is expected to occur more frequently under moderate wind speeds.

We used global positioning system (GPS) telemetry location data from adult female polar bears and modelled surface windscapes to examine the significance olfaction plays in movement patterns, and test the hypothesis that bears move cross-wind during olfactory search. We specifically examined cross-wind olfactory search as our sampling rate was too low (4-hour and 30-minute fix rate) to investigate upwind movement behaviour associated with localization of the source. We predicted that the use of cross-wind olfactory search by polar bears would be more common during winter, at night, and under moderate wind speeds.

## Methods

**Figure 1:**
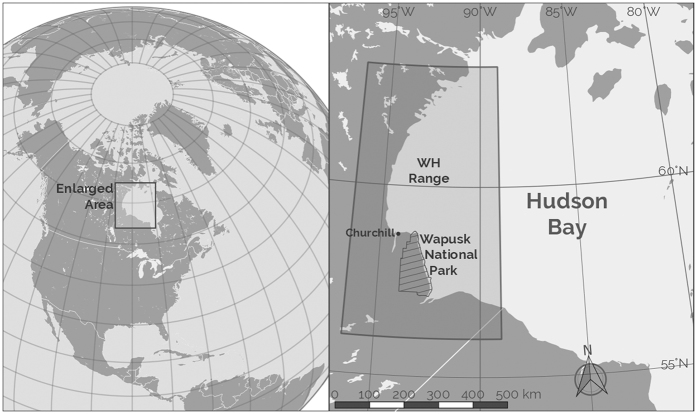
Study area in Hudson Bay, Canada. Shaded area represents the western Hudson Bay (WH) polar bear subpopulation management boundary. Map was created using QGIS version 2.14 ESSEN (http://www.qgis.org/en/site/)^80^.

Hudson Bay, Canada is a large inland sea, which covers an area of 83*10^4^ km^2^ (Fig. 1)^48^ and is seasonally ice-free^49^. From January to early May, it is covered by both fast ice (connected to shore or sea bottom) and drifting pack ice^50^. During break-up (early July), the motile ice drifts southeast as a consequence of the counter-clockwise gyre^51^ and northwesterly winds^52^.

As part of a study of the population ecology of polar bears in western Hudson Bay^44^,^53^,^54^, polar bears were captured during the summers of 2004–2014. Bears were located and captured from helicopters^55^, and a sample of adult females with offspring were fitted with Argos^®^ satellite-linked GPS collars (Telonics, Mesa, AZ). Collars were programmed to last 2 years, and had release mechanisms to drop them on a predefined date. Lone females were not collared as they were possibly pregnant and would remain in maternity dens up to seven months after collaring. Males were not collared because their neck circumference is greater than their head circumference and do not retain collars. Animal handling protocols were approved by the University of Alberta Animal Care and Use Committee for Biosciences and by the Environment Canada Prairie and Northern Region Animal Care Committee. Animal handling and collar deployments performed were in accordance with the approved protocols.

A total of 123 collars were deployed (9–15 per year); most (120) obtained one location every 4 hours, whereas 3 obtained locations every 30 minutes and were analyzed separately. The latitude and longitude coordinates were converted into Universal Trans Mercator coordinate system (NAD83 Teranet Ontario Lambert, EPSG: 5321) in R version 3.2^56^.

Surface wind speeds and directions were modelled by the National Center for Environmental Prediction (NCEP) and obtained from the NOAA Operational Model Archive and Distribution System (NOMADS) (http://nomads.ncdc.noaa.gov/data/gfsanl/)^57^. Biases in the wind direction estimates were identified by comparing model outputs to empirical wind measured at the Churchill Airport, Manitoba (58.74°N, 94.07°W). Empirical wind data at six hour intervals were obtained from http://climate.weather.gc.ca/ (accessed on October 15, 2015).

NCEP generates gridded wind estimates at 6 hour intervals at 1° resolution (approximately 55 km longitude and 111 km latitude). To maximise the fit of wind data to movement data, only locations ≤4 hours apart were used. As the times and coordinates of both wind and movement data were not synchronized, wind data were spatially and temporally interpolated to match coordinates of bear locations. First, the wind was spatially interpolated to the location of the bear using inverse-distance weighting both before and after the time of a bear location^58^. Because wind estimates are both uniformly distributed in space (across a 1° grid) and have low resolution, the four wind estimates adjacent to a bear’s location were used. Second, the two spatial estimates were linearly interpolated to match the time of the location fix.

While on the sea ice, a portion of a bear’s absolute displacement is involuntary and driven by ice drift^41^,^42^. Thus, to study voluntary movement related to wind-associated foraging, the component ice drift was subtracted from the location data^42^. Ice drift data (Polar Pathfinder Daily 25 km EASE-Grid Sea Ice Motion Vectors) were acquired from the National Snow and Ice Data Center^59^. Ice drift was spatially interpolated using inverse distance weighting to match the bear locations^58^. The results of wind bias, and effect of removing ice drift are presented in the supplementary materials under ‘*Wind model and ice drift bias*.’

The data were filtered by four factors that may affect prevalence of cross-wind olfactory search: season, wind speed, bear speed, and presence of daylight. As habitat characteristics change over the year, and likely influence optimal foraging behaviour, we analyzed data separately by season: summer (on-land locations June 1 – October 31), autumn (on-land locations November 1–30), freeze-up (offshore locations November 1 – December 31), winter (offshore locations January 1 – June 30), and break-up (offshore locations July 1 – August 31)^33^. The overlap in the on-land and offshore seasons handles variation in sea ice formation across the Bay and between years, and individual variation in movement phenology.

As wind velocity plays a role in olfactory foraging efficiency, the data were subdivided into “slow” and “fast” wind categories. However, because we had no *a priori* threshold for wind speed at which behaviours change, we tested a moving threshold between 10.8 km/h and 54 km/h and presented the results in the supplementary material (Supplementary Tables S1 to S6). Although the whole range was tested, the presented data used thresholds that were representative of natural breaks in the moving threshold analysis (usually 36 km/h).Different bear speeds may reflect different behaviours (e.g., various olfactory search strategies, travel, rest, prey consumption). The 4-hour resolution of the collars likely includes periods of rest and movement. Additionally, the instantaneous bear velocity at each location may differ from the mean speed between successive locations because any deviations from the straight line path or variable velocities between the successive locations are not captured^60^. To limit analysis to potential olfactory search, we removed what we considered at-rest data (<0.01 km/h). To account for different behaviours and periods of rest data, the remaining movement data were divided into “slow” and “fast” bear speeds at thresholds between 0.5 km/h and 6 km/h between successive locations (Supplementary Tables S1 to S6). As with the wind speed threshold, the presented data used bear speed thresholds representative of natural breaks (usually 2 km/h). For each season, data were grouped into one of four categories: (1) slow wind and slow bears, (2) fast wind and slow bears, (3) slow wind and fast bears, and (4) fast wind and fast bears. Given our prediction of cross-wind search at moderate speeds, we did not expect predominant cross-wind orientation in categories (2) or (4) (due to fast winds). Intervals with slow bear speed were more likely to contain at-rest behaviour or still hunts, and so cross-wind orientation was not expected in categories (1) or (2) (due to slow bears). Thus, cross-wind orientation was predicted to be highest in category (3), slow winds and fast bears. For brevity, categories where predominant orientation relative to wind were similar were pooled and presented together. For example, “fast wind o*r* slow bea*r*s” encompasses all data in categories (1), (2), and (4).

To test whether there was a circadian behavioural pattern, sunrise and sunset times were determined for each coordinate using the ‘sunriset’ function of ‘maptools’ package in R^61^. “Day” and “night” were defined by the sun being above or below the horizon, respectively, at each location.

Predominant bear direction relative to wind direction was assessed using χ^2^ tests. Data were binned into one of five directions: (1) tail winds (<25° between bear and wind bearings), (2) cross-tail winds (≥25° & <65°), (3) cross-wind (≥65° & <115°), (4) cross-head winds (≥115° & <155°), (5) and head winds (≥155° & ≤180°). Under the null hypothesis that bear direction is random with respect to wind direction, the expected ratio among the categories would be 5:8:10:8:5, respectively. These methods are adapted from Spear and Ainley, 1997^62^, Weimerskirch *et al*.^15^, Paiva *et al*.^63^, and Zavalaga *et al*.^64^, which tested for anemotaxis during foraging using movement data relative to wind. Because of the moving thresholds of wind and bear speeds, each movement datum was analyzed within each wind/bear speed category. To control for multiple tests of each data point, a Bonferroni adjustment was made (statistical significance = 0.0006; based on 7 wind speed and 12 bear speed thresholds). If a set of data was statistically significant, adjusted standardized residuals were calculated to identify dominant orientation. All analyses were conducted both on the 4-hour collars and the 30-minute collars. To determine whether any patterns were artefacts of sampling rate, the 30-minute collar locations were sampled at a 4-hour interval by isolating all locations that were 4 hours apart. These results are presented in the supplementary material under ‘*Sampling rate bias*.’ To determine whether autocorrelation affected the observed patterns, we sampled the 4-hour collars by removing all steps <12 hours apart and ran the same χ^2^ tests. These results are presented under ‘E*ffect of autocorrelation.*’ Temporal autocorrelation decay to random patterns as sampling time-lag is enforced^65^. As polar bear behaviours show diurnal patterns^19^ we assume that autocorrelation is negligible after 12 hours.

Means of unimodal distributions were calculated using the ‘mean.circular’ function from ‘circular’ package in R^56^. The two means of bimodal distributions were calculated using the ‘movMF’ package in R, which fits two von Mises-Fisher distributions using maximum likelihood^66^.

## Results

### Movement relative to geographic north

**Figure 2:**
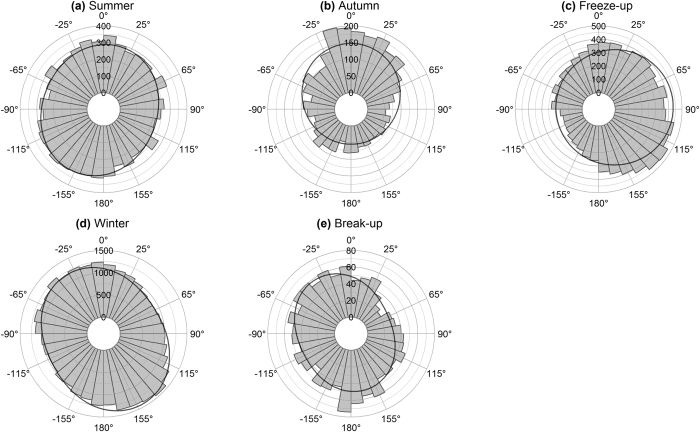
Movement relative to north (0°). Frequency of polar bear orientation during (**a**) summer, (**b**) autumn, (**c**) freeze-up, (**d**) winter, and (**e**) break-up. Curves represent probability density functions based on maximum likelihood of a mixture of two (for **a**, **d** and **e**) and a single (for **b** and **c**) von Mises-Fisher distributions.

During summer, bears exhibited marginal bidirectionality with modes around −152° (SSW) and 15° (NNE) (Fig. 2a). During autumn, predominant movement was 0° (N), with northward movements nearly three times more frequent than eastward, westward, or southward movements (Fig. 2b). During freeze-up, predominant movement was 84° (E) (Fig. 2c). Winter and break-up movement exhibited bimodal distributions with modes around −33° (NNE) and 152° (SSE) (Fig. 2d and e).

### Contribution of ice drift to displacement

During freeze-up and winter, when bears were moving slowly (<2 km/h) or when wind was fast (>36 km/h), bear orientation was unimodal with the mean displacement 20° relative to the wind bearing (Supplementary Fig. S1a). Movement with the component of ice drift removed was a mean −2° relative to the wind bearing (Supplementary Fig. S1b). We expected mean polar bear orientation to be symmetrical relative to the wind direction and not bias orientation toward the left or right of wind. As movement with ice drift removed deviated less from symmetry than without (Supplementary Fig. S1), all subsequent analyses were based on movement with ice drift removed.

### Movement relative to wind

**Figure 3:**
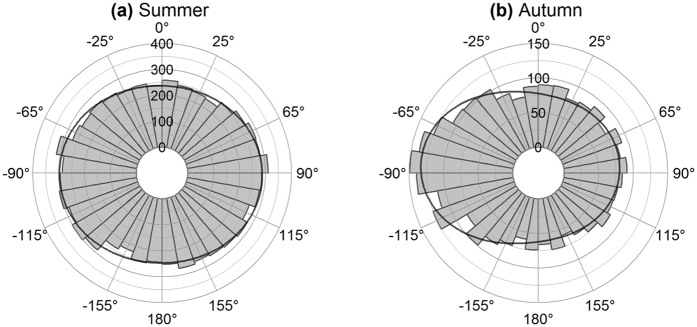
Movement relative to wind on land. Frequency of polar bear orientation during (**a**) summer and (**b**) autumn while wind speed was <36 km/h and polar bear speed was <2 km/h. Curves represent probability density function based on maximum likelihood of a mixture of two von Mises-Fisher distributions.

Predominant orientation of the bears was cross-wind during summer and autumn regardless of wind or bear speeds (Supplementary Tables S1 and S2). While winds were slow (<36 km/h) and polar bear speeds slow (<2 km/h), the two modes of summer movement were at 94° and −90° relative to the wind bearing (Fig. 3a). During autumn, the two modes were at 79° and −85° relative to wind, with the latter being more frequent (Fig. 3b). Although the orientation was statistically significant (summer, χ^2^ = 42, df = 4, P < 0.0001; autumn, χ^2^ = 68, df = 4, P<0.0001), bear orientation was more strongly associated with angle to north (angular dispersion = 0.24) than with wind bearing (angular dispersion = 0.12) (Fig. 2a and b vs. Fig. 3; Kruskal-Wallis χ^2^ = 48, df = 1, P < 0.0001, Wallraff test of angular dispersion).

**Figure 4:**
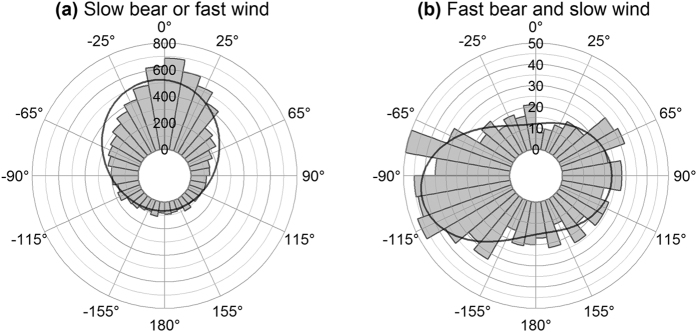
Movement relative to wind during freeze-up. Frequency of polar bear orientation relative to wind while (**a**) polar bear speed was <2 km/h or wind speed was >21.6 km/h and (**b**) polar bear speed was >2 km/h and wind speed was <21.6 km/h. Curves represent probability density functions based on maximum likelihood of a single (for **a**) and a mixture of two (for **b**) von Mises-Fisher distributions.

During freeze-up, the predominant orientation of the bears relative to wind was linked to both polar bear and wind speeds. Slower bear movements (<2 km/h) or movements while wind was fast ( > 21.6 km/h) were predominantly tail wind (Supplementary Table S3; Fig. 4a, χ^2^ = 5002, df = 4, P < 0.0001). Orientation was more strongly associated with angle to wind (angular dispersion = 0.46) than with north (angular dispersion = 0.25) (Fig. 2c vs. Fig. 4; Kruskal-Wallis χ^2^ = 681, df = 1, P < 0.0001, Wallraff test of angular dispersion). Fast polar bear movements (>2 km/h) while wind was slow (<21.6 km/h) were predominantly cross-wind (Supplementary Table S3; Fig. 4b), with modes at 90° and −100° relative to the wind (Supplementary Table S3; Fig. 4b; χ^2^ = 77, df = 4, P < 0.0001), however, only 8% (n = 882) of the freeze-up data fell into this category.

**Figure 5:**
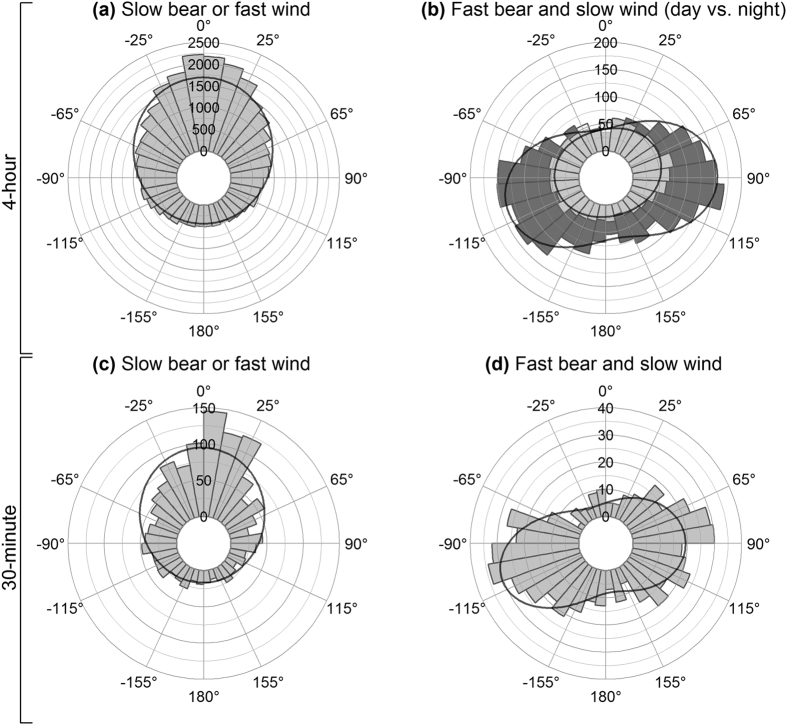
Movement relative to wind during winter. Frequency of polar bear orientation relative to wind while polar bear speed was <2 km/h or wind speed was >36 km/h (**a** and **c** - representing two collar types, see below), and while polar bear speed was >2 km/h and wind speed was <36 km/h (**b** and **d** - representing two collar types, see below). (**a** and **b**) represent collars that had 4-hour fix intervals while (**c** and **d**) represent collars that had 30-minute fix intervals. (**b**) is subset into day (light grey) and night (dark grey). Curves represent probability density functions based on maximum likelihood of a single (for a and c) and a mixture of two (for b and d) von Mises-Fisher distributions.

During winter, slower bear movements (<2 km/h) or movements while wind was fast ( > 36 km/h) were predominantly tail wind (Supplementary Table S4; Fig. 5a; mode = −1 °, χ^2^ = 8520, df = 4, P < 0.0001). Fast polar bear movements (>2 km/h) while wind was slow (<36 km/h) were predominantly cross-wind (Supplementary Table S4; Fig. 5b; mode_1_ = 81°, mode_2_ = −102°, χ^2^ = 275, df = 4, P < 0.0001). Dividing the fast bear and slow wind data into day and night revealed a circadian pattern, with more cross-wind movement at night than during the day (Fig. 5b). As with the 4-hour collars, 30-minute collars exhibited predominantly downwind movement during slow bear movement or under fast winds (Supplementary Table S5; Fig. 5c; mode = −3 °, χ^2^ = 649, df = 4, P < 0.0001), whereas fast bear movements under slow winds were predominantly cross-wind (Supplementary Table S5; Fig. 5d; mode_1_ = 90°, mode_2_ = −109°, χ^2^ = 113, df = 4, P < 0.0001). For the 30-minute data, 26% of winter data fell into the ‘slow wind and fast bear’ category, compared to 10% of the 4-hour winter data.

**Figure 6:**
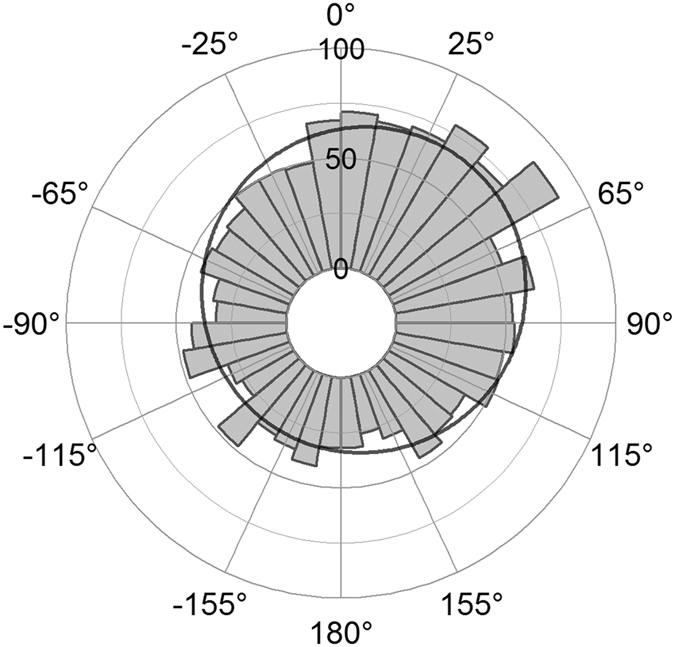
Movement relative to wind during sea ice break-up. Frequency of polar bear orientation relative to wind bearings. Curve represents probability density function based on maximum likelihood of a von Mises-Fisher distribution.

During ice break-up, polar bear movements were predominantly cross-tail wind with a unimodal orientation of 34° relative to the wind, regardless of collar location frequency or bear or wind speeds (Supplementary Table S6; Fig. 6; χ^2^ = 89, df = 4, P < 0.0001). All orientation patterns described above are consistent when subsampled at 12-hour intervals to remove autocorrelation (Supplementary Table S7).

## Discussion

We observed polar bear movement patterns that were associated with season, presence of daylight, wind speed, and bear speed. Seasons vary in food distribution and habitat conditions^34^,^36^,^67^. As food abundance and distribution change and as energetic cost of foraging change, different foraging behaviours may be optimal^32^,^38^,^68^. Within any season, wind speed can influence the effectiveness of olfactory search. Higher wind speeds increase turbulence and affect odour filament distribution and may be unfavourable for olfaction^13^,^46^,^47^. In addition, bear speed might reflect different behaviours, only some of which are cross-wind search^19^. Other behaviours such as travel between patches, migration, rest, or visual search likely exhibit different relationships with wind than cross-wind search.

Distinguishing between behaviours is complicated by the delineation of biologically meaningful seasons and by the resolution and accuracy of the wind estimation, ice drift estimation, and GPS tracking data. Although the modelled wind direction was a mean 10° left of wind measured at Churchill Airport (Supplementary Fig. S2), it falls within the bin sizes used in the χ^2^ tests (±25° or ±20°, depending on orientation), and would not cause misclassification of the predominant direction. The 4-hour resolution of the location data can only capture sustained movements, masking short-term responses to wind, such as upwind localization of a detected odour. Despite the inherent challenges and limitations of studying animals with vast and remote ranges, we observed several wind- and season-associated behaviours.

In Hudson Bay, terrestrial foraging by polar bears is limited to less energetically dense foods (e.g., berries, and seaweed)^34^,^35^,^36^,^37^, and the bears minimize unnecessary movement to conserve energy^32^,^38^,^39^. We found a weak (though significant) association between bear movement and wind during summer, suggesting that cross-wind search is either reduced or absent during this season (Fig. 3a). We found a similar bimodal distribution in movement relative to north, with bears tending to move north or south (Fig. 2a). Any movement during the summer would be confined by the shoreline, which extends north-south in western Hudson Bay (Fig. 1). Because of the predominantly northwesterly winds (Supplementary Fig. S3), random movement confined by the shoreline would tend to also be cross-wind. Thus, the cross-wind movement we observed during summer may be an artefact of the landscape rather than a response to wind.

Freeze-up begins in northwest Hudson Bay^69^ and, by moving northwards during the months leading up to freeze-up, bears are able to return to the sea ice sooner. The northward movement we observed during autumn (Fig. 3b) may be an example of polar bears’ migratory behaviour associated with freeze-up^32^.

Polar bears traveled predominantly downwind during freeze-up (Fig. 4a), which leads them east towards the centre of the Bay (Fig. 2c). In addition to following the southeastward advancing sea ice, we suggest the movement may be partly guided by wind. Because wind direction was variable (Supplementary Fig. S3), movement guided solely by celestial or global cues (such as solar position or global magnetisms)^41^ would have a stronger association relative to north than relative to wind, which was not the case (Fig. 2c vs Fig. 4a). As intraspecific competition affects distribution of female polar bears with cubs^70^, the focus of such bears during freeze-up may be to disperse throughout the Bay and away from conspecifics. The pattern of dispersal during freeze-up may be sex-specific as females avoid males due to the threat of infanticide^33^. However, we cannot draw conclusions regarding male movement, because only females with young were collared.

We observed cross-wind movement during freeze-up, however, only at low wind and high bear speeds (Fig. 4b). Because predominant cross-wind movement during freeze-up was found only at lower wind speeds (<21.6 km/h) compared to winter (<36 km/h), it suggests that some foraging during freeze-up may occur.

During winter, at high wind speeds or when polar bears were moving slowly movement was predominantly downwind (Fig. 5a) and was not predicted. Explanations for the downwind movement include that it may represent a default orientation that generally leads bears southeast and further into the Bay. Second, it may be a thermoregulatory response to high wind speeds; moving downwind minimises the surface area of a bear exposed to wind and shields the face. Thermoregulatory downwind orientation has been modelled and observed for several taxa^71^,^72^. Third, if wind direction fluctuates more than 30° from the mean, then upwind or downwind movement provide more information about the environment than cross-wind movement, a phenomenon described as the geometric pattern of scent dispersion^8^,^13^. However, the geometric pattern of scent dispersion alone cannot account for the low frequency of upwind movement observed. Finally, behaviours apart from cross-wind search, such as travel, still-hunting, movement following habitat features, or other olfactory search strategies may tend to be downwind. Non-olfactory behaviours occupy around 60% of polar bear time budgets^19^,^25^.

During winter, at low wind speeds and while polar bear speeds were high, movement was predominantly cross-wind (Fig. 5b) and matched our predicted movement for cross-wind olfactory search. If the cross-wind movement during freeze-up and winter is reflective of cross-wind olfactory search, the greater frequency at lower wind speeds aligns with findings that polar bear hunting success increases with decreasing wind speed^73^. Cross-wind movement was also more common at night than during the day (Fig. 5b), supporting our hypothesis that movement is primarily guided by olfaction during periods of darkness, while movement during the day may rely, in part, on visual cues. We observed cross-wind movement more frequently among bears wearing collars that had 30-minute fix rates than those wearing collars with 4-hour fix rates (Fig. 5b vs. d). Additionally, the proportion of 30-minute data in the slow wind and fast bear category was 2.6 times greater than among 4-hour collars, suggesting that directionalities observed among the lower resolution collars underestimated the proportion of cross-wind movement. Additionally, the 30-minute data that was subsampled at 4-hour intervals revealed the same patterns as non-subset 30-minute data and 4-hour data, where movement was downwind while winds were fast or bears were slow, and movement was cross-wind while winds were slow and bears were fast (Supplementary Fig. S5), suggesting that the observed patterns were not an artefact of sampling rate.

An alternative explanation for cross-wind movement is that it is a response to environmental features that are associated with the predominant winds, such as pressure ridges^74^. However, environmental features cannot account for the association between bear orientation and wind speed or presence of daylight, as was exhibited during freeze-up and winter. Specifically, the presence of pressure ridges is independent of wind speed and presence of daylight, while cross-wind movement was dependent on these factors. Additionally, because of the counter-clockwise gyre in Hudson Bay and changing winds, pressure ridges in the Bay are not uniformly cross-wind.

During break-up, mean polar bear movement was 34° relative to the wind (Fig. 6). With the predominant northwesterly winds, this would take the bears southeast towards shore and following the retreating ice. The movement relative to north shows a large component of northwestward movement (Fig. 2e), which may reflect bears compensating for ice drift^41^,^42^ before returning to land as hunting conditions deteriorate. As the season progresses and sea ice melts, polar bears may spend increasingly more time swimming, during which collars cannot transmit locations^75^,^76^. As such, limiting analysis to only 4-hour collars does capture the complete range of behaviours, especially during break-up.

We predicted that polar bears employ cross-wind olfactory search and predicted that this would occur more frequently during winter, when they enter hyperphagia^54^, under moderate wind speeds, when conditions are conducive for olfaction 73,77,78, at higher bear speeds, when they are more likely engaged in active search, and at night, when olfaction may be more effective than visual search^45^. The observed cross-wind movement during winter generally supports our hypotheses and predictions. Olfactory foraging may vary across populations due to patterns of sea ice distribution and raises additional research questions. Does maintenance of relative position on drifting ice^41^,^42^ come at the expense of prolonged olfactory search? Polar bears on more stable ice may be less active than bears on drifting ice^38^ - what is the role of olfaction in more stable habitats? Arctic wind speeds are projected to increase due to climate change^79^ and could impede polar bear hunting success^73^. Further studies using higher temporal resolution location data, in combination with direct observation of hunting bears, would further our understanding of olfactory predation. Additionally, given its influence on behaviour, wind could be incorporated as a characteristic in habitat selection modelling of olfactory predators, as the quality of a habitat may be dependent on windscapes. In practice, windscapes could be used as modifiers to the available habitat (e.g., fast winds invoke downwind movement, while cross-wind movement would be favoured under moderate winds). To our knowledge, this is the first such evidence of cross-wind orientation for olfactory search for any wild, non-avian carnivore. The methods presented here are widely applicable and can provide insight on olfactory search among predators across taxa (e.g., canids, felids, and mustelids) and on prey using olfaction to avoid predators.

## Additional Information

**How to cite this article**: Togunov, R. R. *et al*. Windscapes and olfactory foraging in a large carnivore. *Sci. Rep.* **7**, 46332; doi: 10.1038/srep46332 (2017).

**Publisher's note:** Springer Nature remains neutral with regard to jurisdictional claims in published maps and institutional affiliations.

## Supplementary Material

Supplementary Material
