# Corrigendum: Windscapes and olfactory foraging in a large carnivore

**DOI:** 10.1038/srep46968

**Published:** 2018-05-31

**Authors:** Ron R. Togunov, Andrew E. Derocher, Nicholas J. Lunn

Scientific Reports 7: Article number: 4633210.1038/srep46332; published online: 04 12 2017; updated: 05 31 2018

This Article contains errors where the ice drift vectors data were incorrectly labelled. Ice drift vectors data is stored as two orthogonal vectors, the horizontal and vertical components, ‘*u*’ and ‘*v*’. In the EASE-grid projection used for the ice drift, the cardinal directions of ‘*u*’ and ‘*v*’ depend on the longitude. However, in the data acquired by the authors, these components were incorrectly labelled as *easting* and *northing*, making the calculated direction of drift incorrect. Because these drift vectors were used to estimate the voluntary movement of polar bears, the subsequent estimates of bear speed and orientation were inaccurate. All statistics using corrected ice drift have been recalculated and the corrected figures presented below. The changes, however, do not alter the fundamental findings of the study.

In the Results section, under subheading ‘Movement relative to geographic north’,

“During freeze-up, predominant movement was 84° (E) (Fig. 2c). Winter and break-up movement exhibited bimodal distributions with modes around −33° (NNE) and 152° (SSE) (Fig. 2d and e).”

should read:

“During freeze-up, predominant movement was 70° (ENE) (Fig. 2c). Winter and break-up movement exhibited bimodal distributions with modes around −35° (NNE) and 150° (SSE) (Fig. 2d and e).”

In the same section, under subheading ‘Contribution of ice drift to displacement’,

“During freeze-up and winter, when bears were moving slowly (<2 km/h) or when wind was fast (>36 km/h), bear orientation was unimodal with the mean displacement 20° relative to the wind bearing (Supplementary Fig. S1a). Movement with the component of ice drift removed was a mean −2° relative to the wind bearing (Supplementary Fig. S1b). We expected mean polar bear orientation to be symmetrical relative to the wind direction and not bias orientation toward the left or right of wind. As movement with ice drift removed deviated less from symmetry than without (Supplementary Fig. S1), all subsequent analyses were based on movement with ice drift removed.”

should read:

“During freeze-up and winter, when bears were moving slowly (<2 km/h) or when wind was fast (>36 km/h), bear orientation was a mean 18° to the wind bearing before removal of ice drift (Supplementary Fig. S1a). Movement with the component of ice drift removed was a mean 23° relative to the wind bearing (Supplementary Fig. S1b). Removing the component of ice drift did not remove the unexpected asymmetry in the apparent movement relative to wind, suggesting a possible underestimation of ice drift. Nevertheless, all subsequent analyses were based on movement with ice drift removed.”

Correct angular dispersions (AD) and results of Wallraff tests of angular dispersion are presented in Table 1. Patterns, significance, and conclusions remain the same.

In the same section, under subheading ‘Movement relative to wind’,

“For the 30-minute data, 26% of winter data fell into the ‘slow wind and fast bear’ category, compared to 10% of the 4-hour winter data.”

should read:

“For the 30-minute data, 25% of winter data fell into the ‘slow wind and fast bear’ category, compared to 10% of the 4-hour winter data.”

and,

“All orientation patterns described above are consistent when subsampled at 12-hour intervals to remove autocorrelation (Supplementary Table S7).”

should read:

“Orientation patterns during freeze-up and winter are consistent when subsampled at 12-hour intervals, however, during movement during summer and break-up did not show significant orientation relative to wind when subsampled (Supplementary Table S7).”

All dominant orientations relative to wind remain the same for the 4-hour collars, however, corrected modes and corresponding statistics are presented in Table 2.

As a result, the corrected Figures 2–6 appear below as Figures 1, 2, 3, 4, 5 respectively. The figure legends are correct.

In the Discussion section,

“During break-up, mean polar bear movement was 34° relative to the wind (Fig. 6). With the predominant northwesterly winds, this would take the bears southeast towards shore and following the retreating ice.”

should read:

“During break-up, orientation to wind was only observed with the 4-hour collars, suggesting that wind-modulated movement is reduced or absent.”

In the Supplementary Materials accompanying this Corrigendum,

“At the locations on ice, there was a significant association between the angles of ice drift and modelled wind bearing (Rayleigh test, r = 0.73, n = 53856, z = 28769, P < 0.0001), with ice-drift averaging 100° clockwise to the wind bearing (Supplementary Fig. S4).”

now reads:

“At the locations on ice, there was a significant association between the angles of ice drift and modelled wind bearing (Rayleigh test, r = 0.73, n = 51888, z = 27909, P < 0.0001), with ice-drift averaging 12° clockwise to the wind bearing (Supplementary Fig. S4).”

Tables S1 to S12 were updated and, where applicable, the captions were updated to include the correct acronyms (e.g., removing “H, head wind” from Table S1 and adding “C, cross-wind” to Table S6). There were no major changes to observed patterns. None of the changes alter the conclusions.

In the Effect of autocorrelation section,

“All of the patterns observed at 4-hour and 30-minute interval were consistent with the 12-hour subset data (Supplementary Table S7). Specifically, orientation to wind during summer and autumn were predominantly cross-wind (Supplementary Tables S8 and S9), orientation to wind during freeze-up and winter were predominantly tail wind if wind speed was high or bear speed was slow or cross-wind if wind speed was slow and bear speed were fast (Supplementary Tables S10 and S11), and orientation during break-up was predominantly cross-tail wind (Supplementary Table S12).”

now reads:

“Nearly all of the patterns observed at 4-hour and 30-minute interval were consistent with the 12-hour subset data (Supplementary Table S7). Specifically, orientation to wind during autumn was predominantly cross-wind (Supplementary Table S9) and orientation to wind during freeze-up and winter were predominantly tail wind if wind speed was high or bear speed was slow or cross-wind if wind speed was slow and bear speed were fast (Supplementary Tables S10 and S11). However, the 12-hour subset data lost the cross-wind orientation during summer (Supplementary Table S8).”

In the Sampling rate bias section,

“Orientation during freeze-up while winds were slow and movements were fast and during break-up were not statistically significant among the 30-minute collars, however this is likely due to low sample size (Supplementary Table S7).

To determine whether any patterns were artifacts of the sampling rate, 30-minutes collars were subsampled at 4-hour intervals. During winter, slower (<2 km/h) bear movements or movements while wind was fast (>36 km/h) were predominantly tail wind (Supplementary Fig. S5a, Supplementary Table S5, and Supplementary Table S7; mode = 13°, χ^2^ = 127, df = 4, P < 0.0001). Fast polar bear movements (>2 km/h) while wind was slow (<36 km/h) were predominantly cross-wind (Supplementary Fig. S5b, Supplementary Table S5, and Supplementary Table S7; mode_1_ = 81°, mode_2_ = −113°, χ^2^ = 51, df = 4, P < 0.0001). Despite a low sample size (after subsampling the two 30-minute collars by a factor of 8), the key results during winter are fully supported.”

now reads:

“Orientation during autumn, during freeze-up while winds were slow and movements were fast, statistically significant among the 30-minute collars, however this is likely due to low sample size (Supplementary Table S7).

To determine whether any patterns were artifacts of the sampling rate, 30-minutes collars were subsampled at 4-hour intervals. During winter, slower (<2 km/h) bear movements or movements while wind was fast (>36 km/h) were predominantly cross-tail wind (Supplementary Fig. S5a, Supplementary Table S5, and Supplementary Table S7; mode = 35°, χ^2^ = 37, df = 4, P < 0.0001). Fast polar bear movements (>2 km/h) while wind was slow (<36 km/h) were predominantly cross-wind (Supplementary Fig. S5b, Supplementary Table S5, and Supplementary Table S7; mode_1_ = 87°, mode_2_ = −118°, χ^2^ = 61, df = 4, P < 0.0001). Despite a low sample size (after subsampling the two 30-minute collars by a factor of 8), the key results during winter are fully supported.”

Updated statistics reported in this section are presented in Table 3.

Figures S1, S3, S4, and S5 have been updated. The figure legends are correct.

## Supplementary Material

Supplementary Information

## Figures and Tables

**Figure 1:**
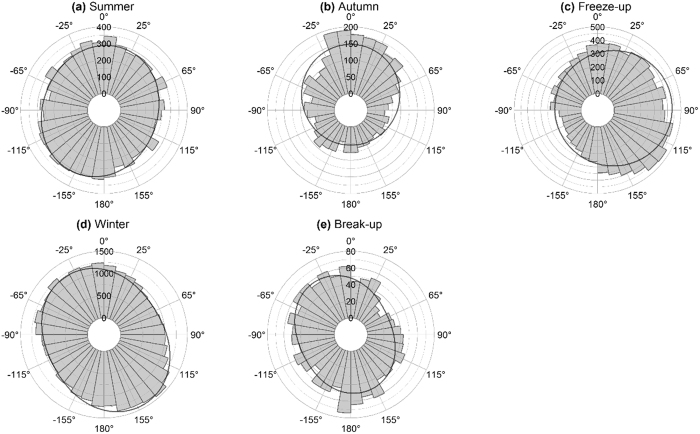


**Figure 2:**
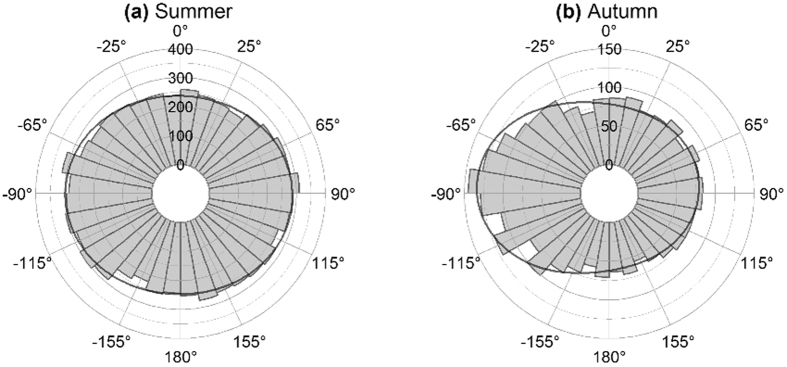


**Figure 3:**
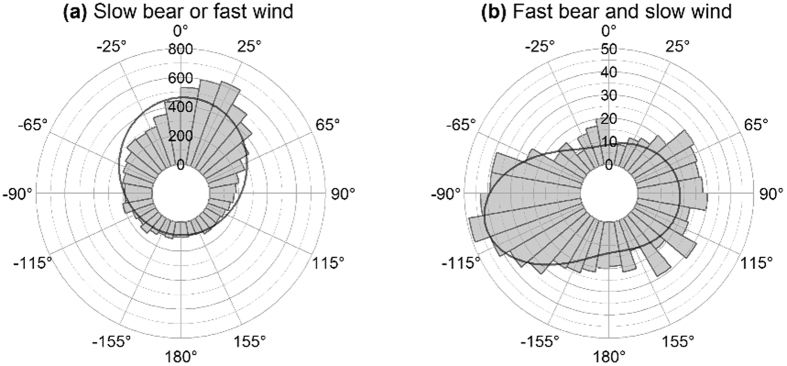


**Figure 4:**
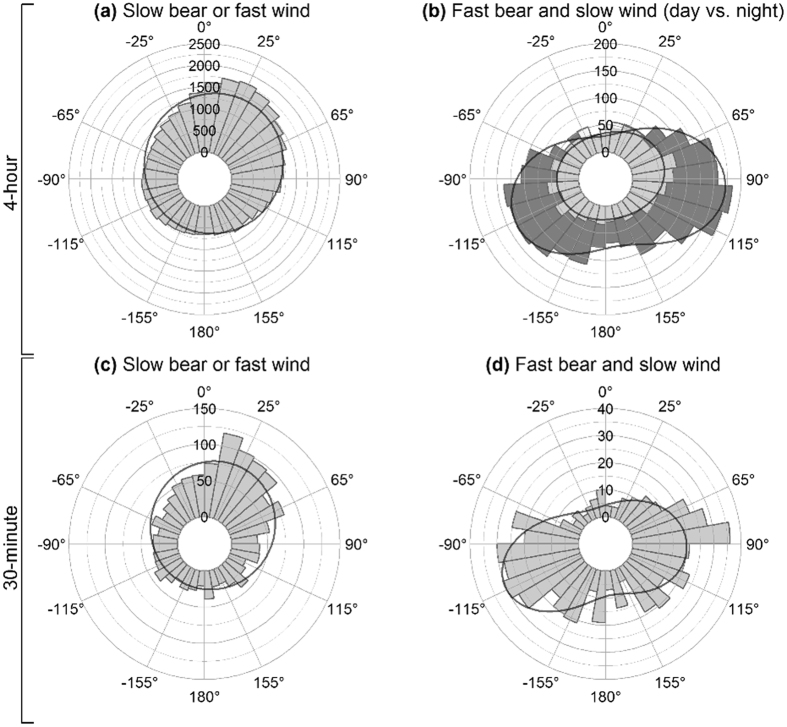


**Figure 5:**
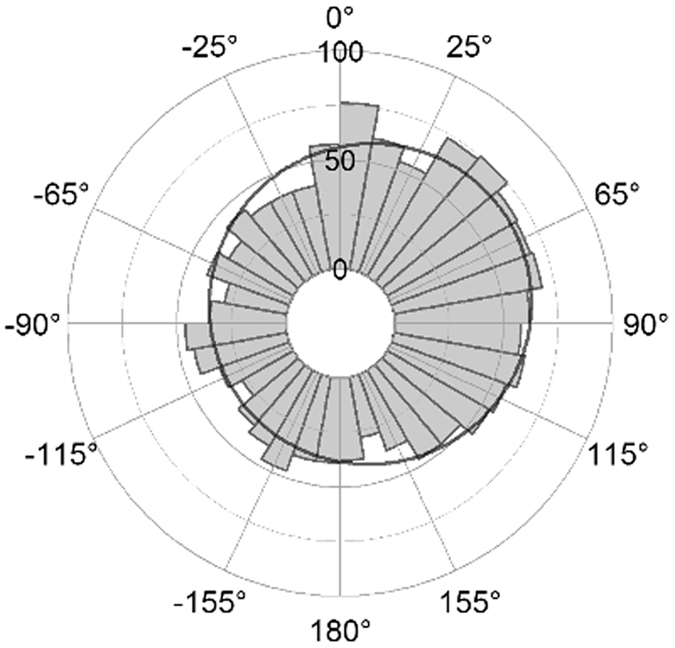


**Table 1:** ^a^Angular dispersion (AD) of polar bear (pb) orientation to wind and North.

Season	Result	AD^a^ (pb-wind vs pb-North)	Kruskal-Wallis χ^2^	df	p-value
Autumn	Reported	0.12 vs. 0.24	48	1	<0.0001
	Updated	0.14 vs. 0.28	23	1	<0.0001
Freeze-up	Reported	0.46 vs. 0.25	681	1	<0.0001
	Updated	0.38 vs. 0.18	577	1	<0.0001

**Table 2:** 

Season	Dataset	Filtering	Result	Modes	n	χ^2^	p-value
Autumn	4-hour	Wind < 36 km/h & PB < 2 km/h	Reported	−85° & 79°	3248	68	<0.0001
			Updated	−87° & 76°	3132	62	<0.0001
Freeze-up	4-hour	Wind > 21.6 km/h or PB < 2 km/h	Reported	−7°	10311	5002	<0.0001
			Updated	5°	10316	3060	<0.0001
		Wind < 21.6 km/h & PB > 2 km/h	Reported	−100° & 90°	882	77	<0.0001
			Updated	−105° & 96°	881	81	<0.0001
Winter	4-hour	Wind > 36 km/h or PB < 2 km/h	Reported	−1°	36454	8520	<0.0001
			Updated	31°	35428	2497	<0.0001
		Wind < 36 km/h & PB > 2 km/h	Reported	−102° & 81°	4128	275	<0.0001
			Updated	−111° & 88°	4158	358	<0.0001
	30-minute	Wind > 36 km/h or PB < 2 km/h	Reported	−3°	1658	649	<0.0001
			Updated	24°	1661	250	<0.0001
		Wind < 36 km/h & PB > 2 km/h	Reported	−109° & 90°	579	113	<0.0001
			Updated	−113° & 93°	547	106	<0.0001
Break-up	4-hour	None	Reported	34°	1737	89	<0.0001
			Updated	58°	1739	35	0.00015

**Table 3:** 

Season	Dataset	Filtering	Result	Modes	direction	χ^2^	p-value
Winter	30-minute subset	Wind > 21.6 km/h or PB < 2 km/h	Reported	13°	Tail wind	127	<0.0001
			Updated	35°	Cross-tail wind	37	<0.0001
		Wind < 21.6 km/h & PB > 2 km/h	Reported	−113° & 81°	Cross-wind	51	<0.0001
			Updated	−118° & 87°	Cross-wind	61	<0.0001

