# Supplementary material for: Corrigendum: Windscapes and olfactory foraging in a large carnivore
Source: Sci Rep. 2018 May 31;8:46968. doi: 10.1038/srep46968 (PMC5977406; doi:10.1038/srep46968)
Supplement: Supplementary Information [file srep46968-s1.doc]

**Supplementary Materials**

**Article title:** Windscapes and olfactory foraging in a large carnivore

**Author list:** Ron R. Togunov, Andrew E. Derocher, and Nicholas J. Lunn

Wind model and ice drift bias


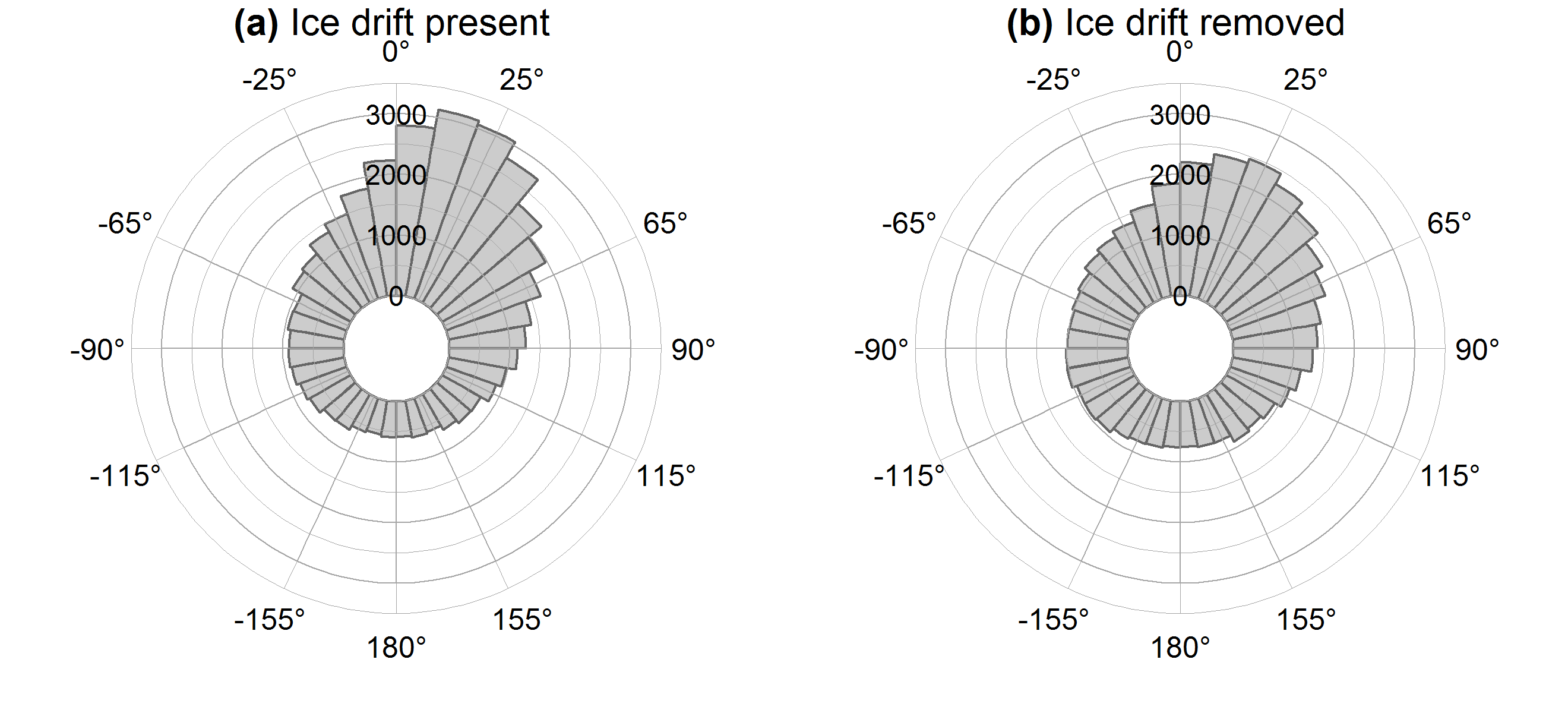


**Supplementary Figure S1.** Frequency of (a) GPS bearing relative to wind and (b) polar bear bearing (with component of ice-drift removed) relative to wind during freeze-up and winter when wind is >36 km/h or polar bear speed is <2 km/h.

The Churchill airport meteorological station has an elevation of 29 m^1^. Although this elevation may not precisely describe ground conditions, we were interested in identifying directional biases in the NCEP model prediction. Within the planetary boundary layer (earth surface up to ~1km), winds are affected by the earth’s surface through friction and the Coriolis Effect^2^. Above ~100m (Ekman layer), wind direction changes due to the Coriolis Effect. However, below ~100m (surface/Prandtl layer), wind speed decreases due to friction but the direction remains almost constant^2^. As the elevation of the weather station is well within the surface layer, we are assuming the meteorological station accurately reflects surface winds direction.

NCEP-modelled wind was a mean 10° anticlockwise to the measured wind bearing at Churchill airport, however, 58% of modelled wind was within ±25° of the bearing measured at the airport (Supplementary Fig. S2).

The bears were exposed to primarily northwesterly winds with mean direction of 134° (SE) (Supplementary Fig. S3). At the locations on ice, there was a significant association between the angles of ice drift and modelled wind bearing (Rayleigh test, r = 0.73, n = 51888, z = 27909, P < 0.0001), with ice-drift averaging 12° clockwise to the wind bearing (Supplementary Fig. S4).

References:

1. *Weather data for: Manitoba, Churchill Ua* http://www.geographic.org/global_weather/manitoba/churchill_ua_606.html (Accessed 2016).
2. Hau, E. *Wind Turbines.* 522-523 (Springer Berlin Heidelberg, 2013).


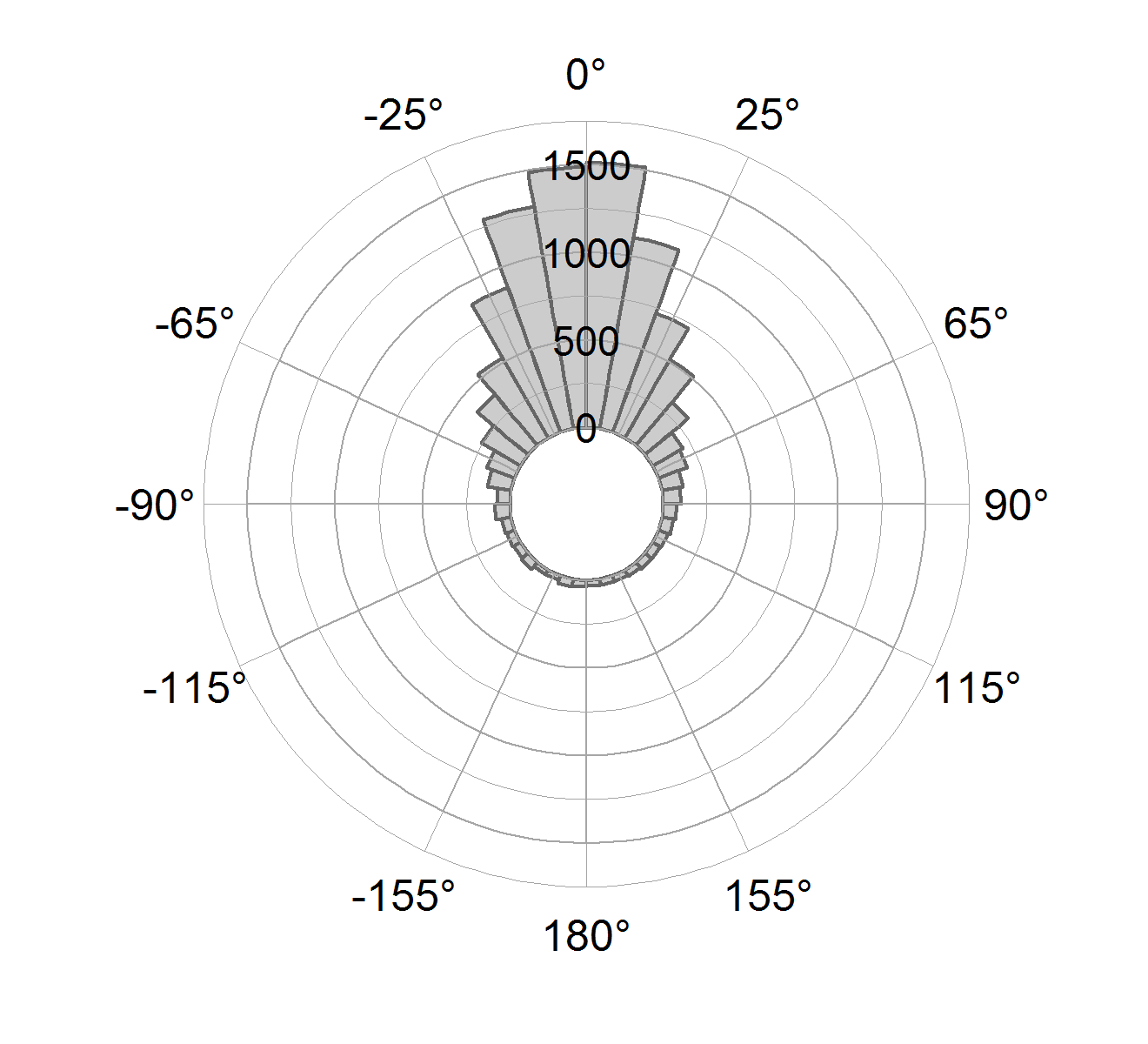


**Supplementary Figure S2.** Frequency plot of angle between modelled wind bearings by NCEP versus measured wind vectors at Churchill airport between September 1, 2004 and April 12, 2012 (n = 11,010).


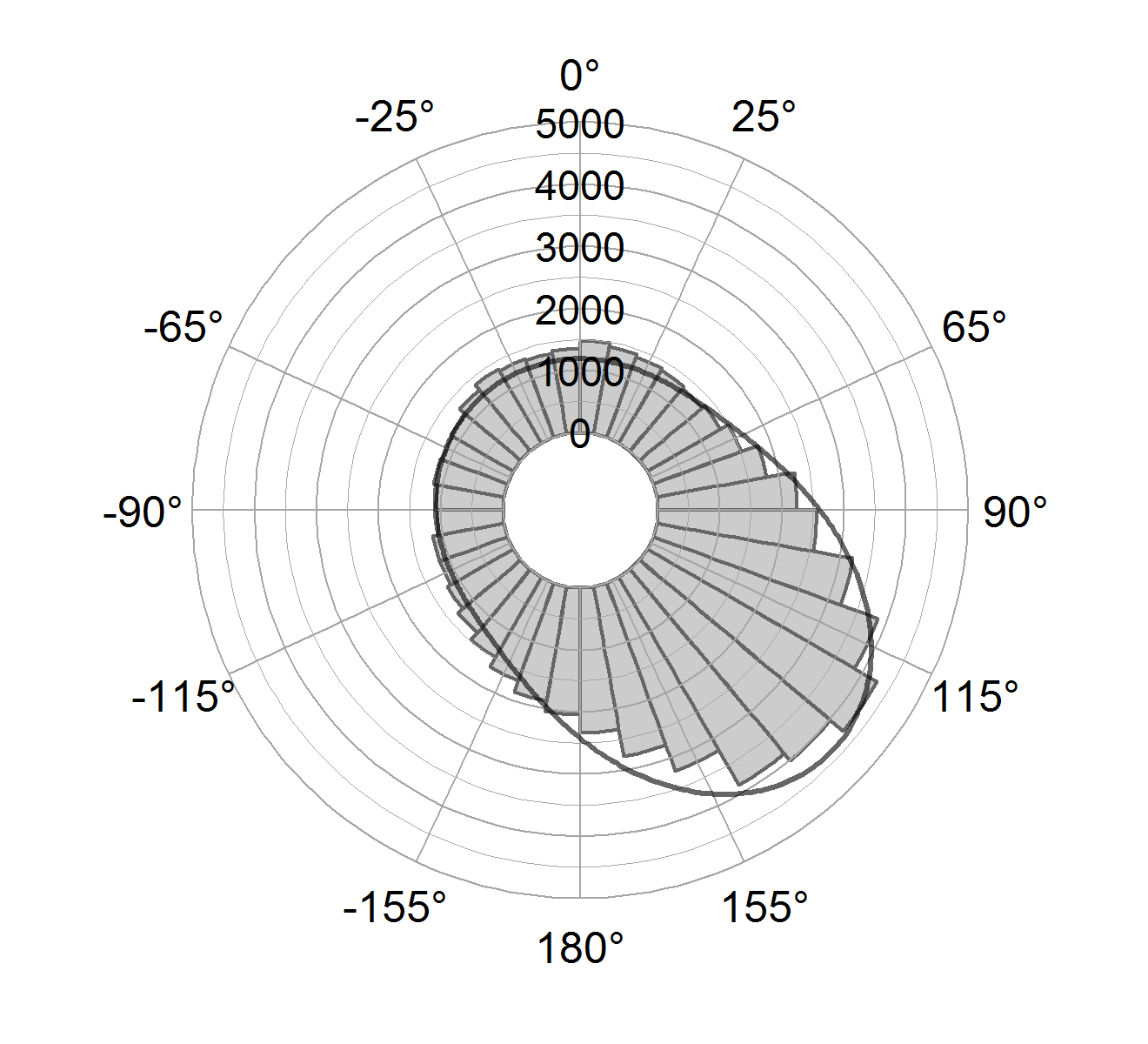


**Supplementary Figure S3.** Frequency plot of wind bearings modelled by NCEP at all bear locations between Sept. 2004 and May 2015. Curve represents probability density function based on maximum likelihood of a mixture of two von Mises-Fisher distributions.


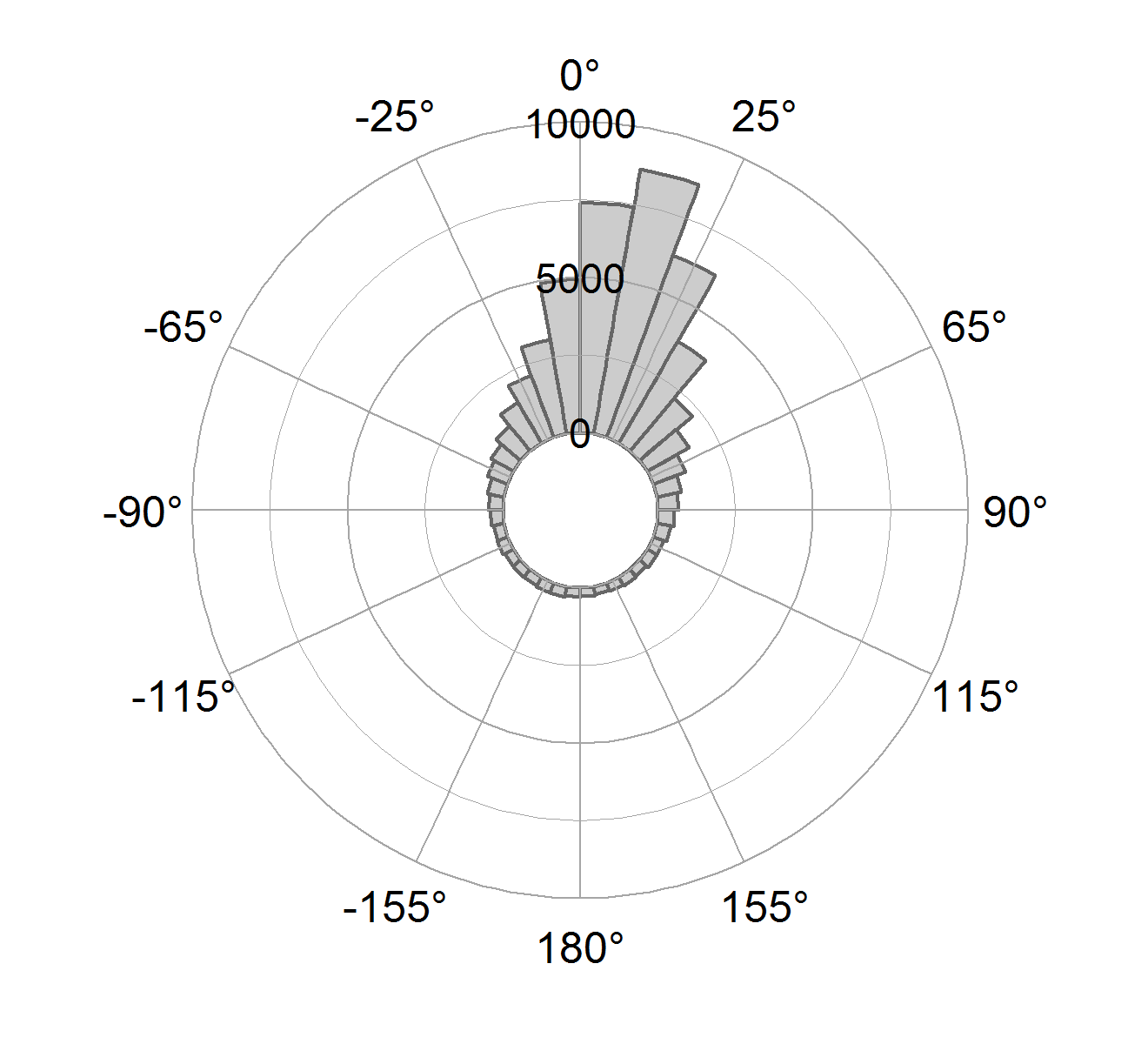


**Supplementary Figure S4.** Frequency plot of acute angle between ice drift and modelled wind bearing at each bear location in Hudson Bay between Sep. 2004 and May 2015.

Tables

The following section contains six tables presenting analysis of significant orientation among movement data divided by wind speed and bear speed at different respective velocities. Each table represents one season and is further subset into four sub-tables that represent: 1) slow wind and slow bear, 2) fast wind and slow bear, 3) slow wind and fast bear, and 4) fast wind and fast bear. Each cell represents the significant orientation of data as or more extreme than the cut-off wind and bear speeds. For example a cell in the second sub-table (fast wind and slow bear) represents the orientation of all the data faster than the wind threshold and slower than the bear threshold. Only statistically significant (alpha value = 0.0006, χ^2^) cells are colour-coded and present the orientation.

To aid in understanding, it is suggested that readers note the dominant colours of the cells in each sub-table to identify key biological patterns.

**Supplementary Table S1. Analysis of bear orientation relative to wind sensitivity to wind and bear speed thresholds during summer.** Greatest adjusted standardized residuals identify dominant orientation: C, cross-wind; CH, cross-head wind; NA, no data; -, not significant (alpha value = 0.0006, χ^2^).

**Supplementary Table S2. Analysis of bear orientation relative to wind sensitivity to wind and bear speed thresholds during autumn.** Greatest adjusted standardized residuals identify dominant orientation: T, tail wind; CT, cross-tail wind; C, cross-wind; NA, no data; -, not significant (alpha value = 0.0006, χ^2^).

**Supplementary Table S3. Analysis of bear orientation relative to wind sensitivity to wind and bear speed thresholds during freeze-up.** Greatest adjusted standardized residuals identify dominant orientation: T, tail wind; CT, cross-tail wind; C, cross-wind; CH, cross-head wind; NA, no data; -, not significant (alpha value = 0.0006, χ^2^).

**Supplementary Table S4. Analysis of bear orientation relative to wind sensitivity to wind and bear speed thresholds during winter.** Greatest adjusted standardized residuals identify dominant orientation: T, tail wind; CT, cross-tail wind; C, cross-wind; CH, cross-head wind; NA, no data; -, not significant (alpha value = 0.0006, χ^2^).

**Supplementary Table S5. Analysis of bear orientation relative to wind sensitivity to wind and bear speed thresholds during winter for collars transmitting at 30 minutes.** Greatest adjusted standardized residuals identify dominant orientation: T, tail wind; CT, cross-tail wind; C, cross-wind; CH, cross-head wind; H, head wind; NA, no data; -, not significant (alpha value = 0.0006, χ^2^).

**Supplementary Table S6. Analysis of bear orientation relative to wind sensitivity to wind and bear speed thresholds during break-up.** Greatest adjusted standardized residuals identify dominant orientation: T, tail wind; CT, cross-tail wind; C, cross-wind; NA, no data; -, not significant (alpha value = 0.0006, χ^2^).

Effect of autocorrelation

To test the effects of autocorrelation on the observed patterns, we subsampled the 4-hour data at by removing all steps <12 hours apart and ran the χ^2^ tests. Temporal autocorrelation decay to random patterns as sampling time-lag is enforced^1^. As polar bear behaviours show diurnal patterns^2^ we assume that autocorrelation is negligible after 12 hours. Nearly all of the patterns observed at 4-hour and 30-minute interval were consistent with the 12-hour subset data (Supplementary Table S7). Specifically, orientation to wind during autumn was predominantly cross-wind (Supplementary Table S9) and orientation to wind during freeze-up and winter were predominantly tail wind if wind speed was high or bear speed was slow or cross-wind if wind speed was slow and bear speed were fast (Supplementary Table S10 and S11). However, the 12-hour subset data lost the cross-wind orientation during summer (Supplementary Table S8). The large number of bears that we are using (123) combined with the relatively low GPS sampling rate remove the majority of effects of autocorrelation generally associated with tracking data.

1. Boyce, M. S. *et al.* Temporal autocorrelation functions for movement rates from global positioning system radiotelemetry data. *Philos. Trans. R. Soc. Lond. B. Biol. Sci.* **365,** 2213–2219 (2010).

2. Stirling, I. Midsummer observations on the behavior of wild polar bears (*Ursus maritimus*). *Can. J. Zool.* **52,** 1191–1198 (1974).

Sampling rate bias

Orientation of the two 30-minute collars match key results of the 4-hour collars including lack of orientation during summer, tail wind movement during freeze-up and winter while winds were fast (>36 km/h) or when bear movement was slow (<2 km/h), and cross-wind movement during winter while winds were slow (<36 km/h) and movement was fast (>2 km/h) (Supplementary Table S7). Orientation during autumn, during freeze-up while winds were slow and movements were fast, statistically significant among the 30-minute collars, however this is likely due to low sample size (Supplementary Table S7).

To determine whether any patterns were artifacts of the sampling rate, 30-minutes collars were subsampled at 4-hour intervals. During winter, slower (<2 km/h) bear movements or movements while wind was fast (>36 km/h) were predominantly cross-tail wind (Supplementary Fig. S5a, Supplementary Table S5, and Supplementary Table S7; mode = 35°, χ^2^ = 37, df = 4, P < 0.0001). Fast polar bear movements (>2 km/h) while wind was slow (<36 km/h) were predominantly cross-wind (Supplementary Fig. S5b, Supplementary Table S5, and Supplementary Table S7; mode_1_ = 87°, mode_2_ = -118°, χ^2^ = 61, df = 4, P < 0.0001). Despite a low sample size (after subsampling the two 30-minute collars by a factor of 8), the key results during winter are fully supported.

**Supplementary Table S7. Bear orientation relative to wind; autocorrelation and sampling rate.** Orientation from 4-hour collars, sampled 4-hour steps >12-hours apart, 30-minute collars, and 30-minute collars sampled at 4-hour interval (alpha value = 0.0006, χ^2^).

| Season | Filtering | Dataset | n | Orientation^a^ | Modes | χ^2^ | DF | P-value |
| --- | --- | --- | --- | --- | --- | --- | --- | --- |
| Summer | Wind <36 km/h &  PB <2 km/h | 4-hour | 9627 | Cross-wind | -90° & 94° | 42 | 4 | <0.0001 |
|  |  | Subset 4-hour | 4740 | Not significant | NA | 12 | 4 | 0.016 |
|  |  | 30-minute | 1024 | Not significant | NA | 7.0 | 4 | 0.138 |
|  |  | Subset 30-minute | 270 | Not significant | NA | 19 | 4 | 0.001 |
| Autumn | Wind <36 km/h &  PB <2 km/h | 4-hour | 3132 | Cross-wind | -87° & 76° | 62 | 4 | <0.0001 |
|  |  | Subset 4-hour | 1598 | Cross-wind | -87° & 83° | 36 | 4 | <0.0001 |
|  |  | 30-minute | 50 | Not significant | NA | 5.2 | 4 | 0.263 |
|  |  | Subset 30-minute | 63 | Not significant | NA | 6.2 | 4 | 0.188 |
| Freeze-up | Wind >21.6 km/h or PB <2 km/h | 4-hour | 10316 | Tail wind | 5° | 3060 | 4 | <0.0001 |
|  |  | Subset 4-hour | 4004 | Tail wind | 5° | 1079 | 4 | <0.0001 |
|  |  | 30-minute | 444 | Tail wind | 35° | 50 | 4 | <0.0001 |
|  |  | Subset 30-minute | 421 | Not significant | NA | 17 | 4 | 0.002 |
|  | Wind <21.6 km/h & PB >2 km/h | 4-hour | 881 | Cross-wind | -105° & 96° | 81 | 4 | <0.0001 |
|  |  | Subset 4-hour | 363 | Cross-wind | -110° & 103° | 54 | 4 | <0.0001 |
|  |  | 30-minute | 59 | Not significant | NA | 3.5 | 4 | 0.481 |
|  |  | Subset 30-minute | 10 | Not significant | NA | 3.1 | 4 | 0.549 |
| Winter | Wind >36 km/h or PB <2 km/h | 4-hour | 36428 | Tail wind | 31° | 2497 | 4 | <0.0001 |
|  |  | Subset 4-hour | 14266 | Tail wind | 33° | 893 | 4 | <0.0001 |
|  |  | 30-minute | 1661 | Tail wind | 24° | 250 | 4 | <0.0001 |
|  |  | Subset 30-minute | 1438 | Cross-tail wind | 35° | 37 | 4 | <0.0001 |
|  | Wind <36 km/h &  PB >2 km/h | 4-hour | 4158 | Cross-wind | -111° & 88° | 358 | 4 | <0.0001 |
|  |  | Subset 4-hour | 1836 | Cross-wind | -114° & 90° | 227 | 4 | <0.0001 |
|  |  | 30-minute | 547 | Cross-wind | -113° & 93° | 106 | 4 | <0.0001 |
|  |  | Subset 30-minute | 144 | Cross-wind | -118° & 87° | 61 | 4 | <0.0001 |
| Break-up | None | 4-hour | 1739 | Cross-tail wind | 58° | 35 | 4 | <0.0001 |
|  |  | Subset 4-hour | 692 | Not significant | NA | 12 | 4 | 0.016 |
|  |  | 30-minute | 283 | Not significant | NA | 6.7 | 4 | 0.150 |
|  |  | Subset 30-minute | 192 | Tail wind | -7° | 40 | 4 | <0.0001 |

^a^Greatest adjusted standardized residuals identify dominant orientation

**Supplementary Table S8. Analysis of 4-hour collars subsampled to 12 hours bear orientation relative to wind sensitivity to wind and bear speed thresholds during summer.** Greatest adjusted standardized residuals identify dominant orientation: C, cross-wind; NA, no data; -, not significant (alpha value = 0.0006, χ^2^).

**Supplementary Table S9. Analysis of 4-hour collars subsampled to 12 hours bear orientation relative to wind sensitivity to wind and bear speed thresholds during autumn.** Greatest adjusted standardized residuals identify dominant orientation: C, cross-wind; NA, no data; -, not significant (alpha value = 0.0006, χ^2^).

**Supplementary Table S10. Analysis of 4-hour collars subsampled to 12 hours bear orientation relative to wind sensitivity to wind and bear speed thresholds during freeze-up.** Greatest adjusted standardized residuals identify dominant orientation: T, tail wind; CT, cross-tail wind; C, cross-wind; CH, cross-head wind; NA, no data; -, not significant (alpha value = 0.0006, χ^2^).

**Supplementary Table S11. Analysis of 4-hour collars subsampled to 12 hours bear orientation relative to wind sensitivity to wind and bear speed thresholds during winter.** Greatest adjusted standardized residuals identify dominant orientation: T, tail wind; CT, cross-tail wind; C, cross-wind; CH, cross-head wind; H, head-wind; NA, no data; -, not significant (alpha value = 0.0006, χ^2^).

**Supplementary Table S12. Analysis of 4-hour collars subsampled to 12 hours bear orientation relative to wind sensitivity to wind and bear speed thresholds during break-up.** Greatest adjusted standardized residuals identify dominant orientation: T, tail-wind; CT, cross-tail wind; NA, no data; -, not significant (alpha value = 0.0006, χ^2^).





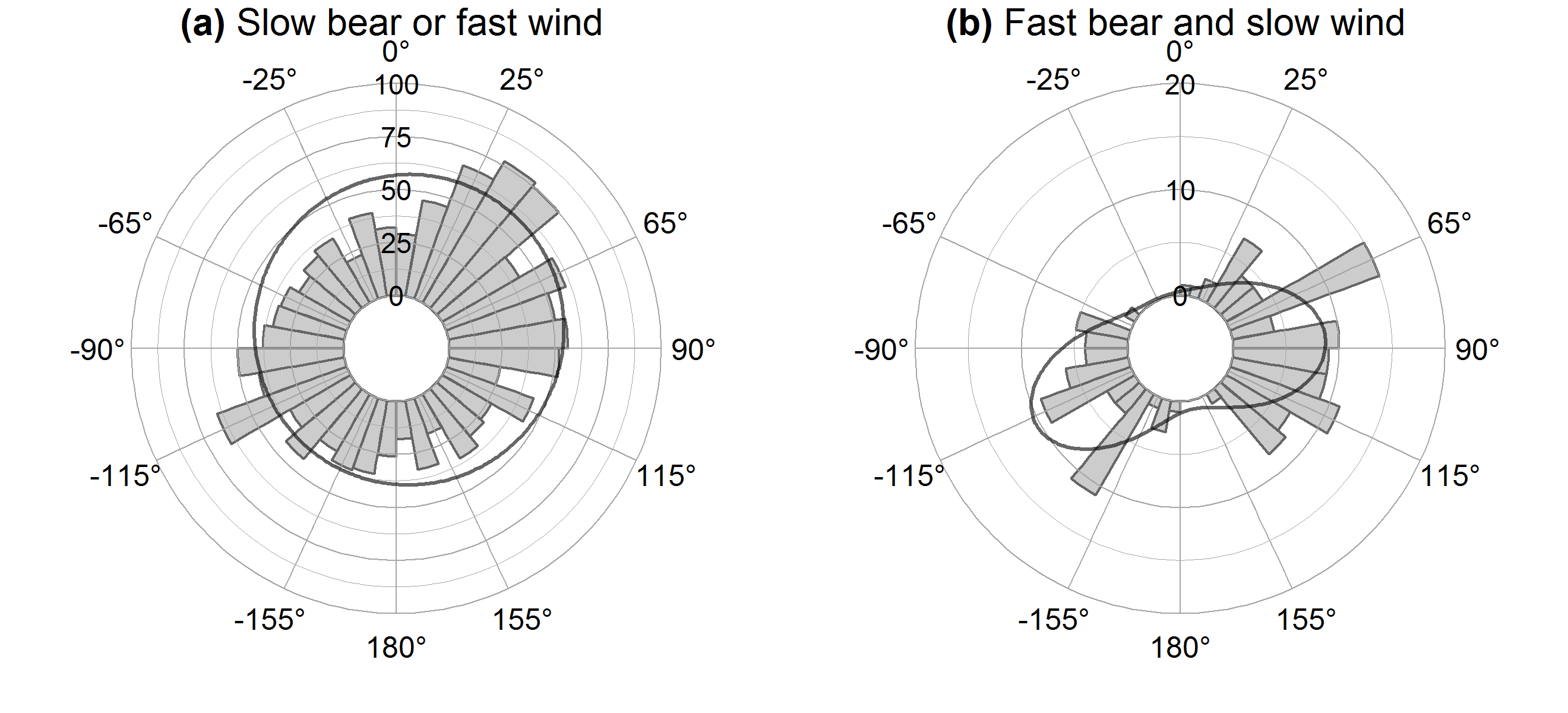


**Supplementary Figure** **S5. Movement relative to wind during winter among subset 30 minute collars.**  Frequency of polar bear orientation relative to wind while (a) polar bear speed was <2 km/h or wind speed was >36 km/h, and (b) while polar bear speed was >2 km/h and wind speed was <36 km/h. Data were from high resolution collars, which had 30-minute fix rate, that were subsampled at 4 hour intervals. Curves represent probability density functions based on maximum likelihood of a single (for a) and a mixture of two (for b) von Mises-Fisher distributions.
